# Digital vs. physical ear-nose-and-throat specialist assessment screening for complicated hearing loss and serious ear disorders in hearing-impaired adults prior to hearing aid treatment: a randomized controlled trial

**DOI:** 10.3389/fdgth.2023.1182421

**Published:** 2023-06-08

**Authors:** Lene Dahl Siggaard, Henrik Jacobsen, Dan Dupont Hougaard, Morten Høgsbro

**Affiliations:** ^1^ Department of Otorhinolaryngology, Head and Neck Surgery, and Audiology, Aalborg University Hospital, Aalborg, Denmark; ^2^ Department of Clinical Medicine, Aalborg University, Aalborg, Denmark

**Keywords:** digital assessment, hearing loss (MesH term), screening, hearing-impaired adults, hearing aid

## Abstract

**Introduction:**

This study introduces a digital assessment tool for asynchronous and remote ear-nose-and-throat (ENT) specialist assessment screening for complicated hearing loss and serious ear disorders in hearing-impaired adults prior to hearing aid (HA) treatment. The +60 population will nearly double from 12% to 22% between 2015 and 2050 increasing the incidence of age-induced hearing impairment and the need for hearing rehabilitation. If un-diagnosed, age-related hearing loss negatively affects quality of life by accelerating social distancing and early retirement as well as increasing risk of anxiety, depression, and dementia. Therefore, innovative measures are essential to provide timely diagnostics and treatment.

**Methods:**

A total of 751 hearing-impaired adults without previous HA usage or experience were randomly assigned to digital or physical ENT specialist assessment screening prior to HA treatment initiation in 20 public and private hearing rehabilitation and ENT specialist clinics in the North Denmark Region. A total of 501 test group participants were assigned to digital assessment screening and 250 control group participants to physical assessment screening prior to HA treatment.

**Results:**

In all, 658 (88%) participants completed the trial and were eligible for analysis. Digital screening sensitivity (0.85, 95% confidence interval (CI) 0.71–0.94) was significantly higher than physical screening sensitivity (0.2, 95% CI: 0.03–0.56). Screening specificity was high for both assessment methods.

**Discussion:**

In a setting where hearing-impaired adults were assessed for HA treatment, digital ENT specialist assessment screening did not compromise patient safety or increase the risk of misdiagnosis in patients with complicated hearing loss and/or serious ear disorders when compared to physical ENT specialist assessment screening.

**Clinical Trial registration:**

https://clinicaltrials.gov/ct2/show/NCT05154539, identifier: NCT05154539.

## Introduction

Hearing loss (HL) negatively affects quality of life by accelerating social distancing and early retirement and by increasing the risk of anxiety, depression, and dementia (1, 2). However, timely and adequately adjusted hearing rehabilitation may counter these negative effects (3). Unfortunately, Danes with age-related HL are at risk of treatment delay due to organizational healthcare system inefficiency and data system misalignment between private and public hearing rehabilitation clinics. Waiting lists for hearing aid (HA) treatment in public healthcare are long, examinations are repeated unnecessarily, and consumers are confused and unaware of their rights because legislation is complex (4). As demographic forecasts predict an increase in the population aged more than 60 years of age from 12% to 22% between 2015 and 2050 (5), the expected number of hearing-impaired individuals will grow and the need for hearing rehabilitation will become more extensive (6). Consequently, hearing healthcare as we know it may fail to accommodate the need for future hearing rehabilitation.

Before acquiring a HA in a private or public hearing rehabilitation clinic, hearing-impaired Danes without prior HA usage or experience need a physical ear-nose-and-throat (ENT) specialist assessment (PESA) screening for complicated HL and/or serious ear disorders that need specialized hospital treatment. This practice is in line with current national guidelines and legislation (7, 8); however, this leaves some hearing-impaired individuals at a disadvantage as current practice prolongs diagnostics and may delay treatment. To remedy such shortcomings, innovative approaches are needed that may enhance treatment flexibility and efficiency and improve socioeconomic resource allocation within the hearing rehabilitation healthcare. One such innovative approach is the digital and remote ENT specialist assessment (RESA) screening method presented in this study. The RESA screening method allows ENT specialists to assess hearing-impaired individuals digitally and remotely without having to consult the patient directly, thereby speeding up the assessment process while avoiding prolonged diagnostic and treatment delays.

The RESA screening method has been tested in 751 individuals in a randomized controlled trial coined “The Innovation of Hearing rehabilitation and Effects of Reform (InHEAR) trial” in the North Denmark Region. The trial aimed to investigate whether RESA screening can be performed in hearing-impaired adults prior to treatment without misdiagnosing cases of complicated HL and serious ear disorders. The aim of this study is firstly to present primary outcomes from the InHEAR-trial: RESA vs. PESA screening accuracy for complicated HL and serious ear disorders in hearing-impaired adults, and secondly to discuss whether RESA screening is a safe and feasible method for digital assessment of hearing-impaired adults prior to HA treatment in the future. Secondary trial outcomes regarding self-reported HA benefit and patient satisfaction in HA recipients undergoing RESA screening will be reported elsewhere.

## Methods

### Trial design and oversight

The InHEAR trial was an open label, randomized controlled trial with two arms and a random 2:1 allocation ratio. Participants allocated to the RESA intervention arm were randomized into two sub-groups: Test group 1 (TG1) and Test group 2 (TG2). TG1 participants were examined and treated in private hearing rehabilitation clinics; TG2, in public hearing rehabilitation clinics. The intervention methodology was identical in the two test groups. In the second arm, control group (CG) participants were assessed physically and in-person by private ENT specialists in accordance with current practice and existing Danish guidelines (7). The trial course comprised three stages: Stage 1, intervention (i.e., examination and in-person/remote ENT specialist assessment); Stage 2, treatment and/or additional examination; and Stage 3, physical gold standard ENT specialist assessment.

### Patient and public involvement

Patient organization representatives, public and private hearing healthcare professionals and collaborators, and representatives from the hearing aid industry were enrolled in a supportive trial committee and participated in finalizing the trial design and selecting the outcome measures prior to trial commencement. Semi-annual meetings enabled committee members to provide critical comments and questions throughout the trial course.

### Participants

Adult individuals aged 18 years or more with subjective HL or hearing difficulties and without associated acute or chronic ear-related symptoms such as earache, ear pain, or discharge from the ears were eligible for participation. Previous HA users and individuals who were unable to understand or read Danish, with severe dementia, or with massive comorbidity that would render impossible their participation, consent, completion of the study questionnaire, or physical attendance at one or more trial stages were not eligible for participation.

A participant registration form was published on a Facebook page maintained by the North Denmark Region via a link to an online survey software where personal data and contact information of interested and potential participants were registered (i.e., full name, home address, phone number, and e-mail address). Subsequently, eligible candidates who met the inclusion criteria received a phone call by one of two study secretaries or a key study administrator who offered additional information about study participation. Clinical study data were obtained throughout the trial by project staff members (e.g., secretaries, audiology assistants, hearing consultants, and ENT specialists) in 12 private and five public hearing rehabilitation clinics and in three private ENT specialist clinics located in the North Denmark Region. Study data were collected and managed using a Research Electronic Data Capture system (REDCap®) hosted at Aalborg University Hospital (9, 10).

### Interventions

At Stage 1, CG participants were examined and assessed physically and in-person by private ENT specialists in accordance with current national guidelines (11). In contrast, TG1 and TG2 participants were examined physically by experienced audiology assistants in private or public hearing rehabilitation clinics, respectively, and then assessed digitally and remotely by ENT specialists. The physical TG examination comprised a standardized test package containing the following three elements: (1) a medical history focused on the ears and hearing, (2) an audiological examination, and (3) an objective examination of the external auditory canal (EAC) and tympanic membrane performed with a digital otoscope.

#### Medical history focused on the ears and hearing

The standardized test package was performed exclusively on TG1 and TG2 participants undergoing RESA screening. The medical history was obtained using the Danish adapted electronic version of the Consumer Ear Disease Risk Assessment (CEDRA) questionnaire (12); a tool designed to assist adult first-time HA users with self-screening for 104 targeted ear diseases before acquiring HAs (13). The questionnaire contains 15 items related to hearing, balance, tinnitus, general health, and other potentially co-occurring symptoms to HL such as vision impairment and recurring fever episodes. Furthermore, the questionnaire produces a score in the 0–28 range, predicting the risk of disease that requires medical attention. The higher the score the higher the risk of having one or more serious ear diseases that require medical attention prior to or in conjunction with HA treatment. The developers of the original tool recommend using a self-screening cut-off score of four (13). To attain an optimal balance between the sensitivity and specificity of the tool in a RESA model setting, the digital ENT specialist assessors were advised to consider the probability of serious ear disorders in TG participants at scores of eight or higher (13). For participants with self-reported tinnitus, the perceived tinnitus handicap severity was measured using the Danish adapted version of the self-reported Tinnitus Handicap Inventory (THI) (14); a 25-item questionnaire with a scoring system ranging from 0 to 100 where higher scores represent a greater perceived tinnitus handicap severity. Participants with THI scores of 58 or higher (severe or catastrophic tinnitus handicap severity) were automatically regarded as complicated cases. Participants with THI scores of 56 or lower (moderate, mild, or slight tinnitus handicap severity) were, in some cases, also categorized as complicated based on information from the additional test results or a supplementary physical consultation with an ENT specialist.

#### Audiological examination

Audiometry test settings, performance, and equipment matched the required standards as described in the Danish Executive Order on HA treatments (8). The test included air and bone conduction thresholds, a speech discrimination test, acoustic reflex tests, and a standard 226 Hz tympanometry test.

#### Video otoscopy

Still images of the EAC and the tympanic membrane were obtained digitally by video otoscopy. Although digital video-otoscopic imaging is becoming a more widely known and accepted diagnostic tool for ear diseases among general practitioners and ENT specialists (15–17), digital video otoscopy was not commonly performed in the participating private and public hearing rehabilitation clinics before their participation in the trial. Therefore, educational examination performance guidelines were composed and distributed among examinators, and test result quality criteria were specified. To counter the complications of earwax blockage, all participants were instructed verbally and in writing to perform a safe earwax removal routine at home or with assistance from their general practitioner before being examined.

#### Digital ENT specialist assessment

The standardized test package results were assessed digitally, remotely, and individually by four experienced ENT specialists: two ENT specialists with medical audiology expertise and two private ENT specialists. All TG participants were randomly allocated to one of the four digital ENT specialist assessors whose assessment decision was subsequently applied at the treatment stage. The participants were blinded to assessment allocation as were the remote ENT specialist assessors.

### Treatment at Stage 2

#### Normal hearing

A pure-tone average (PTA) hearing level of 20 dB or better was regarded normal. PTA referred to the average air conduction (AC) hearing thresholds at 500, 1,000, 2,000, and 4,000 Hz (PTA-4). To ensure that serious ear disorders were not overlooked in the intervention groups, TG participants with normal hearing undergoing RESA at Stage 1 were re-examined physically by an ENT specialist with medical audiology expertise before concluding their trial course.

#### Simple HL

Simple HL included HL in the subcategories mild (25–40 dB hearing level AC thresholds) and moderate (45–60 dB hearing level AC thresholds) without concurrent serious ear disorders. Participants with asymmetric sensorineural mild or moderate HL of 15 dB or more at two neighboring frequencies were offered additional magnetic resonance imaging (MRI) of the internal auditory canal to rule out retro-cochlear pathology such as a vestibular schwannoma. Brain stem audiometry (ABR) was applied in case of MRI contraindications. TG1 participants with simple HL had HAs fitted in private hearing rehabilitation clinics; TG2 participants, in public hearing rehabilitation clinics. CG participants with simple HL were allowed to choose between a private or public hearing rehabilitation clinic for HA fitting. Regardless of group affiliation, all participants were treated in line with current national clinical guidelines on HL management (7).

#### Complicated HL and serious ear disorders

Complicated HL included symmetric and asymmetric HL exceeding the 61 dB hearing level AC thresholds and mild or moderate HL with severe PTA-4 asymmetry exceeding 30 dB in AC hearing thresholds between the two ears and/or a difference of 20% or more in speech discrimination score (DS) between the two ears. Complicated HL was diagnosed in accordance with the 2015 Danish National Clinical Guideline (NCG) criteria on ENT specialist assessment and referral of patients with HL (7). The translated list of criteria is presented in Table 1. Serious ear disorders comprised: (1) EAC pathology (e.g., atresia, exostosis, otitis externa, cholesteatoma of the EAC), (2) middle ear pathology (e.g., cholesteatoma, otosclerosis, tympanic membrane perforation or retraction, infection, and secretory or acute otitis media), (3) retro-cochlear pathology (e.g., vestibular schwannoma, tinnitus, otogenic vertigo), and (4) cerebral pathology (e.g., infection, tumor, head trauma, vascular disorders, neurological issues). Regardless of group affiliation, all participants with complicated HL and/or serious ear disorders were referred to the Department of Audiology at Aalborg University Hospital for an additional physical ENT specialist assessment before initiating treatment.

**Table 1:** The 2015 Danish National Clinical Guideline Criteria for ENT specialist assessment and referral of patients with hearing loss.

Types of patients with complicated HL that require specialized medical ENT specialist assessment at an audiology hospital department
• All patients below 18 years of age* • Patients in need of assessment and treatment defined as a regional and highly specialized hospital function in accordance with current guidelines • Patients with significantly reduced speech-reception thresholds regardless of the extent of their hearing loss, corresponding to a speech discrimination score (DS) < 75% measured by speech audiometry (Dantale I) • Patients with asymmetric hearing loss, where the averaged asymmetry in hearing thresholds at 500, 1,000, 2,000, and 4,000 Hz is more than 30 dB, and/or where the difference in speech DS between the two ears is 20 or higher. Further assessments to disregard retrocochlear disease may still be indicated at averaged asymmetries below 30 dB • Patients in whom a hearing aid is considered for an ear with an average hearing of 25 dB hearing level or better at 500, 1,000, 2,000, and 4,000 Hz • Patients who may be candidates for cohclear implants, bone-anchored hearing aids, or other implantable hearing aid solutions. • Patients with hearing loss and concomitant severely bothersome tinnitus and patients with severely bothersome tinnitus without hearing loss • Patients with hearing loss in combination with other severe sensory impairment and/or complicating comorbidity and/or severely reduced functional capacity of importance for the treatment of choice* • Patients with fluctuating or rapidly progressive hearing loss

ENT, Ear-nose-and-throat; DS, Discrimination score; HL, Hearing loss.

* Because of study exclusion criteria, these patient categories were not represented in the study population.

### In-person gold standard assessment at Stage 3

Regardless of group affiliation, all participants diagnosed with HL (simple and complicated) or serious ear disorders were re-assessed by experienced ENT specialists subspecialized in medical audiology or otology at the Department of Audiology at Aalborg University Hospital 2–4 months after initiating HA treatment. A 30-minute, in-person patient-physician consultation was conducted and a oto-microscopy was performed by the ENT specialist for objective assessment of the EAC and the tympanic membrane. This physical ENT specialist re-assessment at Stage 3 served as the gold standard with which all previous remote or in-person Stage 1 ENT specialist assessments were compared.

### Outcomes

Primary outcome measures comprised RESA vs. PESA screening sensitivity and specificity of TG and CG participants, respectively, for complicated HL and serious ear disorders. Remote and in-person ENT specialist assessments at Stage 1 in TG and CG participants, respectively, were compared to physical gold standard ENT specialist assessments at Stage 3, and screening sensitivity and specificity were calculated and analyzed for TG1, TG2, and CG participants, respectively. All ENT specialist assessments at Stages 1 and 3 were thoroughly cross reviewed and revised by two key project management members in case of obvious registration errors or project guideline misconceptions.

### Sample size

RESA screening applicability in hearing-impaired individuals depends on how accurately remote ENT specialist assessors identify cases of complicated HL and serious ear disorders. However, some ear disorders are rare such as cholesteatoma with 350 new cases (5–10 per 100,000 individuals) and vestibular schwannoma with 200 new cases (4 per 100,000 individuals) diagnosed yearly in Denmark (18). The incidence of otosclerosis with clinical manifestations are 6–14 per 100,000 individuals in Europe (18). The minimal sample size required to test for RESA sensitivity and specificity for these conditions would be substantial; and trial execution would be extremely costly, time-consuming, and practically impossible. Instead, the RESA method aimed to screen for a combined group of individuals with complicated HL and/or serious ear disorders where incidences of cholesteatoma, vestibular schwannoma, and other ear disorders were presumed to be higher. The prevalence of complicated HL and/or serious ear disorders in Danish hearing-impaired adults was estimated to be 5% based on a statistical analysis on functional hearing measures in hearing rehabilitation in Denmark from 2000 to 2006 published by the National Board of Social Services in 2010 (19). More recent national statistical inventories are not available on the distribution of HL subcategories in adult hearing-impaired individuals.

According to the literature, the minimum required sample size for determining the sensitivity and specificity of a screening test depends on the pre-specified values of power, the corresponding level of type 1 error, and the effect size (20). As the prevalence of complicated HL and/or serious ear disorders was estimated to 5%, a minimum of 400 individuals were required to achieve a minimum power of 80% for detecting a change in the percentage value of sensitivity of a screening test from 0.50 before conducting the study to 0.80 after conducting the study based on a *P* value of 0.05 (20). Thus, 400 TG participants were included along with an additional 100 individuals to accommodate a 20% drop-out risk. To add a statistical basis of comparison between patients undergoing RESA and PESA, an additional 250 CG participants were included. As PESA screening sensitivity and specificity were expected to be higher than the corresponding RESA values, the required CG sample size was smaller.

### Randomization

Participants who had received and digitally signed a consent form were eligible for randomization. A random allocation sequence was generated with statistical software R v4.1.2 (21) using the R package “REDCapAPI” v2.5.0 (22) and uploaded to the randomization module by a key administrator. To minimize selection and accidental bias and ensure balanced allocation of participants, block randomization was applied. A block size of 12 was divisible by the total number of groups (TG1, TG2, and CG) and provided 12 possible factorial ways of assigning participants to a block while eliminating the risk of repeat blocks. No stratification variables were applied. The allocation sequence was uploaded to REDCap® and concealed from all study personnel including investigators, field staff, and participants. Thus, prediction or deciphering of participant allocation was not possible. Randomization was completed by two study secretaries who were given user access to the randomization tool.

### Statistical methods

RESA screening sensitivity and specificity in the TG groups were calculated by comparing remote ENT specialist assessments at Stage 1 with in-person gold standard ENT specialist assessments at Stage 3 in two times tables using Fischer's exact test. Similarly, PESA screening sensitivity and specificity in CG participants were calculated by comparing physical ENT specialist assessments at Stage 1 with the in-person gold standard ENT specialist assessments at Stage 3. All measures were presented with 95% confidence intervals (CI), and a *P* < 0.05 was considered statistically significant. All statistical tests were performed with statistical software R v4.1.2.

## Results

**Figure 1:**
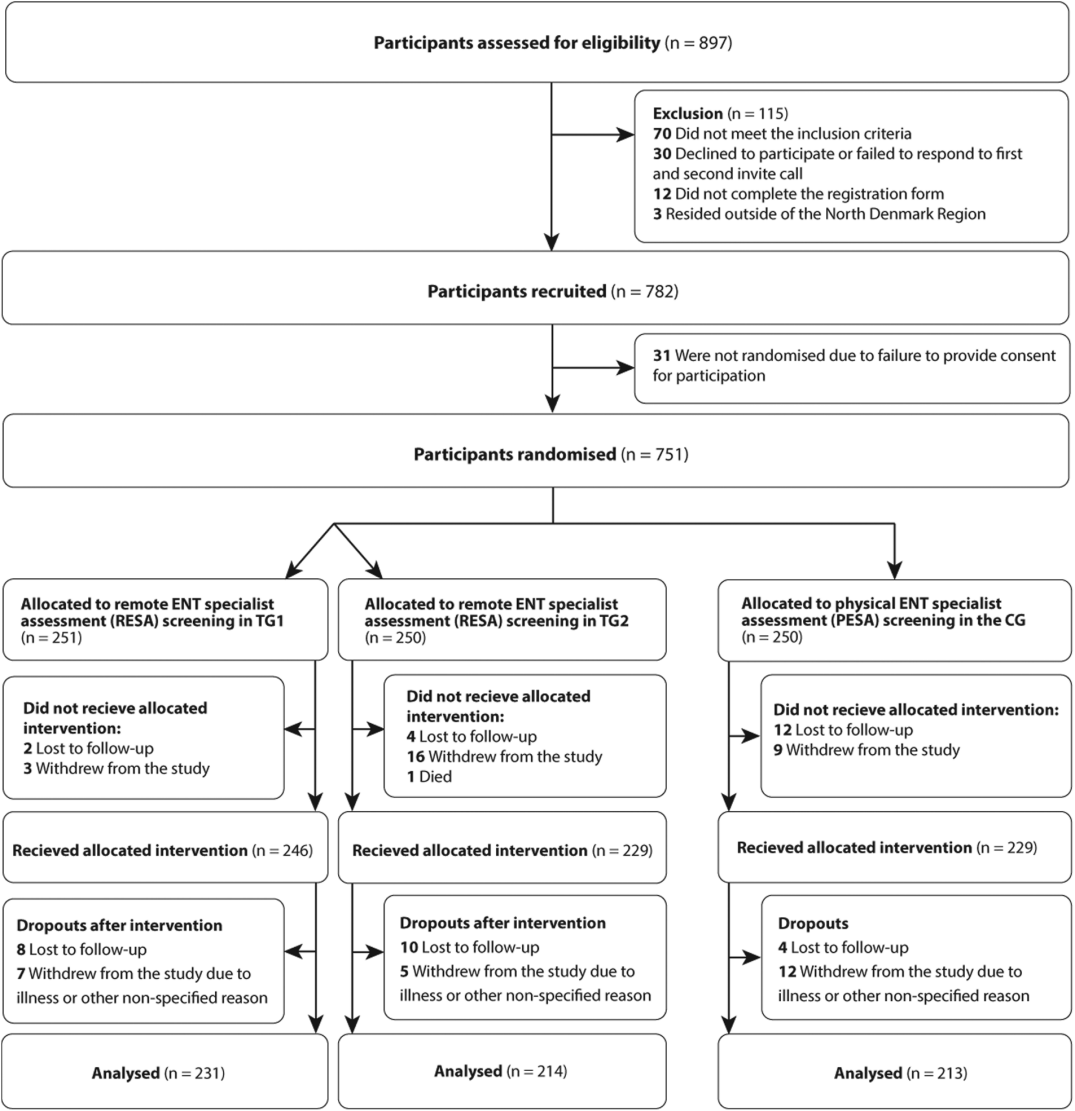
CONSORT flow diagram. HA, Hearing aid; ENT, Ear, nose and throat; RESA, Remote ENT specialist assesment; PESA, Physical ENT specialist assesment; TG1, Test group 1; TG2, Test group 2; CG, Control group.

A total of 782 individuals aged 18 years or more with subjective HL were recruited between March, 2021, and September, 2021. The trial was completed in May 2022. A total of 751 participants signed the informed consent form and were eligible for randomization, and 658 (88%) participants completed the trial and were eligible for analysis. A total of 40 (5%) participants were lost to follow-up, 52 (7%) participants withdrew from the study due to illness, or for personal or unknown reasons, and one participant died during the trial course. Participant flow is shown in Figure 1.

No significant difference was found in age and gender composition between the groups (Table 2). Out of the 658 participants who completed the study, 457 (69%) participants were offered HAs. Among these, 50 (11%) participants (25 participants in TG1, 15 participants in TG2, and 10 participants in the CG group) were diagnosed as “complicated” at Stage 3: two cases of cholesteatomas, three cases of otosclerosis, nine cases of conductive HL with suspected otosclerosis, three cases of tympanic membrane perforation, two cases of otitis media with effusion (OME), one case of eustachian tube dysfunction (ETD), five cases of other middle ear or auditory canal pathology (e.g., myringo-incudo-pexy, postoperative radical cavity, EAC cholesteatoma or skin pathology), eight cases of severely bothersome tinnitus, and 17 cases of complicated asymmetric HL.

**Table 2:** Baseline characteristics. Values are numbers (percentages) unless otherwise stated.

		Randomized study population (*N* = 751)	
Baseline characteristics	Test group 1 (*n* = 251)	Test group 2 (*n* = 250)	Control group (*n* = 250)
Mean (SD) age, years	63	63	62
Female sex of participants	136 (54)	139 (56)	131 (52)
		Analyzed study population (*N* = 658)	
Type of HL according to gold standard assessment	(*n* = 231)	(*n* = 214)	(*n* = 213)
No HL/normal hearing	67 (29)	60 (28)	74 (35)
Simple HL	140 (61)	137 (64)	129 (60)
Complicated HL and/or serious ear disorder	24 (10)	17 (8)	10 (5)

SD, Standard deviation; HL, Hearing loss.

Table 3 presents the RESA and PESA screening sensitivity and specificity in TG and CG participants, respectively. The table shows four possible outcomes when comparing RESA/PESA screening at Stage 1 to the ENT specialist gold standard assessments at Stage 3. The sensitivity is the proportion of participants with complicated HL and/or serious ear disorders that were correctly identified as such in the TGs and the CG, respectively. The specificity is the proportion of participants with normal hearing or simple HL that were correctly identified as such in the TGs and the CG, respectively. The positive predictive value (PPV) of RESA/PESA screening is the probability that participants diagnosed with complicated HL and/or serious ear disorders at Stage 1 truly have either complicated HL and/or one or more serious ear disorder. The negative predictive value (NPV) of RESA/PESA screening is the probability that participants diagnosed with normal hearing or simple HL at Stage 1 truly have either normal hearing or simple HL. For RESA screening, sensitivity was 0.85 (95% CI: 0.71–0.94), specificity was 0.97 (0.95–0.98), and the PPV and NPV were 0.74 (0.60–0.86) and 0.98 (0.97–0.99), respectively, *P* = 0.006. For PESA screening, sensitivity was 0.20 (0.03–0.56), specificity was 1.00 (0.97–1.00), and the PPV and NPV were 0.67 (0.09–0.99) and 0.96 (0.93–0.98), respectively, *P* < 0.001. A sub analysis on RESA screening accuracy in TG1 and TG2 participants is shown in Table 4. For RESA screening in TG1, sensitivity was 0.88 (0.68–0.97), specificity was 0.98 (0.95–0.99), and the PPV and NPV were 0.84 (0.64–0.95) and 0.99 (0.96–1.00), respectively, *P* = 0.006. For RESA screening in TG2, sensitivity was 0.82 (0.57–0.96), specificity was 0.96 (0.92–0.98), and the PPV and NPV were 0.64 (0.41–0.83) and 0.98 (0.96–1.00), respectively, *P* < 0.001.

**Table 3:** RESA vs. PESA screening sensitivity and specificity in TG and CG participants, respectively.

	Physical gold standard ENT specialist assessment at Stage 3		
	Complicated HL and/or serious ear disorders	Simple HL and/or normal hearing	
Remote ENT specialist assessment (RESA) at Stage 1 (TG1 + TG2)			Total
Complicated HL and/or serious ear-related condition	35	12	47
Simple HL and/or normal hearing	6	392	398
**Total**	41	404	445
Physical ENT specialist assessment (PESA) at Stage 1 (CG)			Total
Complicated HL and/or serious ear-related condition	2	1	3
Simple HL and/or normal hearing	8	202	210
**Total**	10	203	213

RESA, Remote ENT specialist assessment; PESA, Physical ENT specialist assessment; ENT, Ear-nose-and-throat; TG, Test group; TG1 Test group 1; TG2, Test group 2; CG, Control group; HL, Hearing loss.

**Table 4:** Sub-analysis of RESA screening sensitivity and specificity in TG1 and TG2 participants.

	Physical gold standard ENT specialist assessment at Stage 3		
	Complicated HL and/or serious ear disorders	Simple HL and/or normal hearing	
Remote ENT specialist assessment (RESA) at Stage 1, TG1			Total
Complicated HL and/or serious ear-related condition	21	4	25
Simple HL and/or normal hearing	3	203	206
**Total**	24	207	231
Remote ENT specialist assessment (RESA) at Stage 1, TG2			Total
Complicated HL and/or serious ear-related condition	14	8	22
Simple HL and/or normal hearing	3	189	192
**Total**	17	197	214

RESA, Remote ENT specialist assessment; ENT, Ear-nose-and-throat; TG, Test group; TG1, Test group 1; TG2, Test group 2; HL, Hearing loss.

### Participants misdiagnosed at Stage 1

Six TG and eight CG participants undergoing RESA and PESA screening, respectively, were misdiagnosed as “simple” at Stage 1 when compared to the gold standard ENT specialist assessment at Stage 3 (Table 5). Twelve TG participants and one CG participant were misdiagnosed as “complicated” at Stage 1 when compared with the gold standard ENT specialist assessment at Stage 3: eight cases of asymmetric HL and five cases of mildly or moderately bothersome tinnitus.

**Table 5:** Test group and control group participants with complicated HL who were misdiagnosed at Stage 1.

Case number	Study group	Diagnosis at Stage 1 (RESA or PESA)	Diagnosis at Stage 3 (gold standard)	Examination/treatment at Stage 3 or later
**TG1 participants with complicated HL or serious ear disorders who were misdiagnosed by RESA screening at Stage 1 (*n* = 3)**				
1	TG1	Simple HL with minor asymmetry and mildly bothersome tinnitus	Complicated HL due to moderate perceived tinnitus handicap (THI grade 3)	• ABR performed to exclude retrocochlear pathology (ABR was normal) • Referral to the IVHD for counselling and tinnitus management guidance
2	TG1	Simple potentially noise-induced HL and moderately bothersome tinnitus	Complicated (not noise-induced) HL due to moderate perceived tinnitus handicap (THI grade 3)	• Referral to the IVHD for counselling and tinnitus management guidance
3	TG1	Simple HL	Complicated mixed sensioneural and minor conductive HL vaguely suspicious for otosclerosis	• The patient was offered otologist assessment but declined
**TG2 participants with complicated HL or serious ear disorders who were misdiagnosed by RESA screening at Stage 1 (*n* = 3)**				
4	TG2	Simple potentially noise-induced HL	Complicated (not noise-induced) HL due to bilateral severely reduced speech discrimination	• Hearing aid administration
5	TG2	Simple HL and moderately bothersome tinnitus	Complicated HL due to moderate perceived tinnitus handicap (THI grade 3)	• Referral to the IVHD for counselling and tinnitus management guidance
6	TG2	Simple potentially noise-induced HL and mildly bothersome tinnitus	Complicated (not noise-induced) HL due to hyperacusis and severe tinnitus problems despite a low perceived tinnitus handicap score (THI grade 2)	• ENT specialist re-assessment due to hyperacusis and tinnitus • Referral to the IVHD for counselling and tinnitus management guidance
**CG participants with complicated HL or serious ear disorders who were misdiagnosed by PESA screening at Stage 1 (*n* = 8)**				
7	CG	Simple HL	Pars flaccida retraction pocket suspicious for cholesteatoma	• Explorative tympanotomy revealed pars flaccida cholesteatoma
8	CG	Simple HL	Pathology of the EAC suspicious for infection and/or malignancy	• Topical ciprofloxacin and dexamethasone combination ear drops • Fast-track biopsy by otologist showed skin infection of the auditory canal and chronic non-malignant inflammation • ENT specialist re-assessment
9	CG	Simple HL	Skin impression of the EAC with epithelial debris but intact coverage of the bony surface, suspicious for early stage of EAC cholesteatoma	• Thorough oto-microscopic epithelial debris clearance and inspection of skin impression • ENT specialist re-assessment for clinical follow-up
10	CG	Simple HL	Complicated HL due to moderate tinnitus problems (THI not completed)	• Audiological re-examination • ENT specialist re-assessment • Referral to the IVHD for counselling and tinnitus management guidance
11	CG	Simple HL	Complicated HL due to severe tinnitus problems (THI not completed)	• Referral to the IVHD for counselling and tinnitus management guidance
12	CG	Simple HL	Conductive HL that requires audiological re-examination and ENT specialist re-assessment due to insufficient audiogram quality	• Audiological re-examination • ENT specialist re-assessment • Final diagnosis: Simple conductive HL not suspicious for otosclerosis
13	CG	Simple HL	Conductive HL that requires audiological re-examination and ENT specialist re-assessment due to insufficient audiogram quality	• Audiological re-examination • ENT specialist re-assessment • Final diagnosis: Simple conductive HL not suspicious for otosclerosis
14	CG	Simple HL	Audiogram not assessable due to insufficient audiogram quality	• Audiological re-examination • ENT specialist re-assessment • Final diagnosis: Simple unilateral HL

RESA, Remote ear-nose-and-throat specialist assessment; PESA, Physical ear-nose-and-throat specialist assessment; TG1, Test group 1; TG2, Test group 2; CG, Control group; HL, Hearing loss; ENT, Ear-nose-and-throat; THI, Tinnitus Handicap Inventory; IVHD, Institute for Vision and Deaf blindness; ABG, Air bone gap.

### Participants with complicated HL and/or serious ear disorders

Another 34 TG participants with complicated HL and/or serious ear disorders were correctly diagnosed at Stage 1, including cases of cholesteatoma, otosclerosis, tympanic membrane perforation, OME, and ETD. In fact, all cases of serious ear disorders that required otologist assessment or intervention in TG participants were correctly assessed by RESA screening at Stage 1.

### Digital otoscopic imaging in TG participants

Out of the 445 TG participants who completed the study, the quality of the digital otoscopic images of the tympanic membranes in 130 (29%) TG participants were considered insufficient by the digital ENT specialist assessors at Stage 1 due to blurring, earwax blockage, or insufficient visualization of the tympanic membrane. In 10 of these cases, the oto-microscopic examination at Stage 3 revealed pathological findings of varying degrees of severity such as earwax blockage, EAC atresia, OME, retraction of the tympanic membrane, and cholesteatoma. However, none of these 10 TG participants were misdiagnosed at Stage 1 when compared to the gold standard ENT specialist assessment at Stage 3.

## Discussion

This randomized trial found that in a setting where hearing-impaired adults were assessed for treatment, overall RESA screening sensitivity for complicated HL and/or serious ear disorders (85%) was significantly higher than PESA screening sensitivity (20%). The screening specificity was high for both screening methods. Thus, RESA screening does not increase the risk of misdiagnoses of patients with HL prior to treatment initiation and could be a potential gamechanger in future ENT assessment practices in hearing healthcare where demographic changes and scarcity of resources may lead to prolonged diagnostics and treatment delay.

Strengths of the study were the large study sample of 751 individuals and the well-organized randomized trial design that eliminated known and unknown confounding variables. A benefit of the public/private cooperation in the study was a cross-sectional RESA method test setting in both private and public healthcare facilities, reflecting the current organization of hearing healthcare in Denmark. Limitations were the lack of blinding of participants and staff. However, the risk of potential experimental biases from participant expectations, from latency between intervention and follow-up stages, and from the influence of other specialist evaluations was considered low. Self-selection bias related to the Facebook-based recruitment strategy must be considered, as volunteering individuals may be younger and healthier, lead healthier lives, and may comply with treatment to a higher degree than older, non-volunteering individuals who may not be as internet literate. Although the 2021 Statistics Denmark report on Danes’ use of information technology shows that 95% of the Danish population between 16 and 74 years were online at least once a day and that 85% were active on social media in 2021 compared with 55% in 2011, social media use seems to decline synchronously with age (23). Thus, elderly people with severe, undiagnosed HL and a lower compliance to treatment may therefore be underrepresented in the present study.

Another element to consider when interpreting the results is the complexity of the underlying pathophysiology of hearing and ear disorders and their various clinical manifestations. As the assessment decision often relies on the individual ENT specialist's experience and professional knowledge and on the influence of the patients’ individual opinions and needs, it may not be possible to define and apply a true ENT specialist gold assessment standard for this patient category. However, in the present study, gold standard assessments were made by experienced ENT specialists subspecialized in either medical audiology or otology. Furthermore, assessments were based on a 30-minute in-person consultation with the patient including an audiological assessment and an objective oto-microscopic examination.

Finally, it is worth mentioning, that Danish PESA-performing private ENT specialists frequently use clinic staff rather than certified audiology assistants to perform audiological examinations on their patients, why the quality of the audiograms may vary significantly, causing an increase in ENT specialist assessment inaccuracy and misdiagnoses. In the present study, three cases of misdiagnosis on this account reduced PESA screening sensitivity from a potential 50% to 20%. To accommodate this type of problems in the future and to specify the quality requirements for hearing rehabilitation in adults, new national guidelines and recommendations have been outlined and will expectedly be released following an upcoming public consultation round.

Asynchronous healthcare technologies utilized in patient portal email messaging, in-app messaging, specialist-to-patient mobile apps, and delayed interview video consultations have been described more frequently in various medical fields such as in radiology, ophthalmology, dermatology, cardiology, pathology, and psychiatry (24–33). In hearing healthcare, technology advances and digital communication options such as hearing apps, tele-audiology, and tele-rehabilitation are also trending and have gained further momentum during the COVID-19 pandemic (34–38). Moreover, the use of artificial intelligence (AI) computer vision algorithms to classify video-otoscopic images has been found to be feasible for classifying ear diseases and enjoy great accuracy (39). In a British study from 2022, 58 adults with HL or tinnitus were reviewed by ENT clinicians using a remote review platform comprising a focused history, audiometric testing, and a smartphone-based application and otoscope (40). The study found an 83.3% diagnosis concordance between remote-review and in-person consultations in 12 patients. However, as described in a review from 2018 concerning eHealth use in the HA adult patient journey, only 10.7% of the observed technology services in hearing healthcare was related to screening and assessment (41). Consequently, a gap remains in the evolution of digital screening technologies used to assess the various modalities deployed for hearing impairment assessment and the complexity of hearing rehabilitation. A novel multivariable digital RESA screening tool for complicated HL and/or serious ear disorders in hearing-impaired adults as presented in the present study is feasible and safe. Moreover, RESA is intended for hearing-impaired individuals with no additional symptoms such as earache, pain, or ear discharge that require ENT specialist assessment and care prior to hearing rehabilitation. For an extensive validation of the tool, regional or national implementation is required.

RESA screening of hearing-impaired adults has great potential in Denmark and in all developed countries where the standard examination routine involves one or more physical encounters with an ENT specialist. RESA simplifies the course of treatment, prevents prolonged diagnostics and treatment delays, and may also improve socioeconomic resource allocation within hearing rehabilitation healthcare without compromising patient safety or lowering the existing examination standards. These benefits are, however, conditioned by the availability of professional hearing care assistants capable of performing valid audiological examinations that meet the required legal quality standards and of conducting high-quality digital otoscopic imaging of the tympanic membrane. Therefore, professional quality requirements and process guidelines on RESA screening of hearing-impaired adults must be outlined to keep quality standards at the needed high level.

## Conclusion

In this randomized trial comprising 751 hearing-impaired adults and potential first-time HA users, we found that RESA screening for complicated HL and/or serious ear disorders did not compromise patient safety. Overall, RESA screening accuracy was significantly higher than the screening accuracy of the physical, in-person ENT specialist assessment routine we know and use today. Moving forward, quality requirements and RESA screening guidelines are needed to ensure the performance of high-quality audiological examinations by professional hearing care assistants. Additional validation and adjustment of the tool is conditioned by regional or national implementation. If these requirements are met, the multivariable RESA screening method presented in this study may revolutionize the screening of hearing-impaired adults in Denmark and potentially also in other developed countries comparable to Denmark.

## Data Availability

The datasets presented in this article are not readily available because owing to Danish legislation, data will be available only after approval by the Danish Data Protection Agency and with a signed access agreement. Requests to access the datasets should be directed to dt@datatilsynet.dk.

## References

[1] Dalton DS Cruickshanks KJ Klein BEK Klein R Wiley TL Nondahl DM. The impact of hearing loss on quality of life in older adults. Gerontologist. (2003) 43:661–8. 10.1093/geront/43.5.661 14570962

[2] Cosh S Helmer C Delcourt C Robins TG Tully PJ. Depression in elderly patients with hearing loss: current perspectives. Clin Interv Aging. (2019):14:1471. 10.2147/CIA.S195824 31616138 PMC6698612

[3] Mahmoudi E Basu T Langa K McKee MM Zazove P Alexander N Can hearing aids delay time to diagnosis of dementia, depression, or falls in older adults? J Am Geriatr Soc. (2019) 67(11):2362–9. 10.1111/jgs.16109 31486068

[4] The Danish Ministry of Health. The future of hearing rehabilitation—a strengthened effort for individuals with hearing loss. Copenhagen, Denmark. (2018). Available at: https://sum.dk/publikationer/2018/oktober/hoereomraadet-i-fremtiden-en-styrket-indsats-for-borgere-med-hoeretab (Cited September 15, 2022).

[5] WHO. WHO: Ageing and health. World Health Organisation. (2021). Available at: https://www.who.int/news-room/fact-sheets/detail/ageing-and-health (Cited September 14, 2022).

[6] GBD 2019 Hearing Loss Collaborators. Hearing loss prevalence and years lived with disability, 1990–2019: findings from the global burden of disease study 2019. Lancet. (2021) 397:996–1009. 10.1016/S0140-6736(21)00516-X 33714390 PMC7960691

[7] The Danish Health Authority. Assessment and referral of patients with hearing loss [national clinical guideline for ear, nose and throat specialists]. Copenhagen, Denmark. (2015).

[8] The Danish Health Authority. Executive order of hearing aid treatment [BEK number 1140 of 11/10/2019]. 1140 Denmark: The Danish Health Authority. (2019). Available at: https://www.retsinformation.dk/eli/lta/2019/1140

[9] Harris PA Taylor R Thielke R Payne J Gonzalez N Conde JG. Research electronic data capture (REDCap)—a metadata-driven methodology and workflow process for providing translational research informatics support. J Biomed Inform. (2009) 42(2):377–81. 10.1016/j.jbi.2008.08.010 18929686 PMC2700030

[10] Harris PA Taylor R Minor BL Elliott V Fernandez M O’Neal L The REDCap consortium: building an international community of software platform partners. J Biomed Inform. (2019) 95:103208. 10.1016/J.JBI.2019.103208 31078660 PMC7254481

[11] The Danish Health Authority. Professional requirement recommendations for hearing aid treatment of adults [national clinical guideline in hearing]. The Danish Health Authority. (2010). Available at: https://hoeringsportalen.dk/Hearing/Details/64253 (Cited September 11, 2022).

[12] Kleindienst SJ Zapala DA Nielsen DW Griffith JW Rishiq D Lundy L Development and initial validation of a consumer questionnaire to predict the presence of ear disease. JAMA Otolaryngol Head Neck Surg. (2017) 143(10):983–9. 10.1001/jamaoto.2017.1175 28772310 PMC5710257

[13] Klyn NAM Robler SK Bogle J Alfakir R Nielsen DW Griffith JW CEDRA: a tool to help consumers assess risk for ear disease. Ear Hear. (2019) 40(6):1261–6. 10.1097/AUD.0000000000000731 30946136 PMC6774904

[14] Zachariae R Mirz F Johansen LV Andersen SE Bjerring P Pedersen CB. Reliability and validity of a danish adaptation of the tinnitus handicap inventory. Scand Audiol. (2000) 29(1):37–43. 10.1080/010503900424589 10718675

[15] Short AB. Efficacy of digital otoscopy in telemedicine [Dissertation]. James Madison University. (2017). Available at: https://commons.lib.jmu.edu/diss201019/157/?utm_source=commons.lib.jmu.edu%2Fdiss201019%2F157&utm_medium=PDF&utm_campaign=PDFCoverPages (Cited September 14, 2022).

[16] Biagio L Swanepoel DW Adeyemo A Hall JW 3rd Vinck B. Asynchronous video-otoscopy with a telehealth facilitator. Telemed J E Health. (2013) 19(4):252–8. 10.1089/tmj.2012.0161 23384332

[17] Lundberg T de Jager L B Swanepoel DW Laurent C. Diagnostic accuracy of a general practitioner with video-otoscopy collected by a health care facilitator compared to traditional otoscopy. Int J Pediatr Otorhinolaryngol. (2017) 99:49–53. 10.1016/j.ijporl.2017.04.045 28688565

[18] Svensson P Ovesen T Buchwald CV Kjeldsen AD Grau C Godballe C Lærebog i øre-næse-hals-sygdomme. 3rd ed. Hammen LN, editor. Copenhagen: Munksgaard. (2022).

[19] Bengtsson S Røgeskov M. People with hearing loss in Denmark. Copenhagen. (2010). Available at: file:///Users/lenesiggaard/Downloads/Personer%20med%20h%C3%B8retab%20i%20Danmark.pdf (Cited September 11, 2022).

[20] Bujang MA Adnan TH. Requirements for minimum sample size for sensitivity and specificity analysis. J Clin Diagn Res. (2016) 10(10):YE01–6. 10.7860/JCDR/2016/18129.8744 27891446 PMC5121784

[21] R Core Team. R: A language and environment for statistical computing. R Foundation for Statistical Computing, Vienna, Austria. (2020). Available at: http://www.r-project.org/index.html (Cited October 5, 2022).

[22] Nutter B Lane S. Accessing data from REDCap projects using the API. (2020). Available at: 10.5281/zenodo.11826 (Cited October 5, 2022).

[23] Tassy A Berg CE. Information technology use in the population 2021. Copenhagen, Denmark. (2022). Available at: dst.dk/da/Statistik/nyheder-analyser-publ/Publikationer/VisPub?cid=39431 (Cited October 19, 2022).

[24] Yellowlees PM Parish MB Gonzalez AD Chan SR Hilty DM Yoo BK Clinical outcomes of asynchronous versus synchronous telepsychiatry in primary care: randomized controlled trial. J Med Internet Res. (2021) 23(7):e24047. 10.2196/24047 33993104 PMC8335606

[25] Chan S Li L Torous J Gratzer D Yellowlees PM. Review of use of asynchronous technologies incorporated in mental health care. Curr Psychiatry Rep. (2018) 20(10):85. 10.1007/s11920-018-0954-3 30155593

[26] Thrall JH. Teleradiology part I. History and clinical applications 1. Radiology. (2007) 243(3):613–7. 10.1148/radiol.2433070350 17517922

[27] High WA Houston MS Calobrisi SD Drage LA McEvoy MT. Assessment of the accuracy of low-cost store-and-forward teledermatology consultation. J Am Acad Dermatol. (2000) 42(5):776–83. 10.1067/mjd.2000.104519 10775853

[28] Shapiro M James WD Kessler R Lazorik FC Katz KA Tam J Comparison of Skin Biopsy Triage Decisions in 49 Patients With Pigmented Lesions and Skin Neoplasms Store-and-Forward Teledermatology vs Face-to-Face Dermatology. Arch Dermatol. (2004) 140:525–8. 10.1001/archderm.140.5.525 15148095

[29] Rotvold GH Knarvik U Johansen MA Fossen K. Telemedicine screening for diabetic retinopathy: staff and patient satisfaction. J Telemed Telecare. (2003) 9(2):109–13. 10.1258/135763303321327984 12699582

[30] Hooper GS Yellowlees P Marwick TH Currie PJ Bidstrup BP. Telehealth and the diagnosis and management of cardiac disease. J Telemed Telecare. (2001) 7(5):249–56. 10.1258/1357633011936471 11571078

[31] Mahnke CB Mulreany MP Inafuku J Abbas M Feingold B Paolillo JA. Utility of store-and-forward pediatric telecardiology evaluation in distinguishing normal from pathologic pediatric heart sounds. Clin Pediatr (Phila). (2008) 47(9):919–25. 10.1177/0009922808320596 18626106

[32] Williams S Henricks WH Becich MJ Toscano M Carter AB. Telepathology for patient care: what am I getting myself into? Adv Anat Pathol. (2010) 17(2):130–49. 10.1097/PAP.0b013e3181cfb788 20179435

[33] Giansanti D Castrichella L Giovagnoli MR. Telepathology requires specific training for the technician in the biomedical laboratory. Telemed e-Health. (2008) 14(8):801–7. 10.1089/tmj.2007.0130 18954250

[34] Jämsä T Israsena P D’onofrio KL Zeng FG. Tele-Audiology: current state and future directions. Front Digit Health. (2022) 1:788103. 10.3389/fdgth.2021.788103 PMC8784511 35083440

[35] Bright T Pallawela D. Validated smartphone-based apps for ear and hearing assessments: a review. JMIR Rehabil Assist Technol. (2016) 3(2):e13. 10.2196/rehab.6074 28582261 PMC5454564

[36] Corona AP Ferrite S Bright T Polack S. Validity of hearing screening using hearTest smartphone-based audiometry: performance evaluation of different response modes. Int J Audiol. (2020) 59(9):666–73. 10.1080/14992027.2020.1731767 32134341

[37] de Sousa KC Moore DR Smits C Swanepoel DW. Digital technology for remote hearing assessment—current Status and future directions for consumers. Sustainability. (2021) 13(18):10124. 10.3390/su131810124

[38] Almufarrij I Dillon H Dawes P Moore DR Yeung W Charalambous AP Web- and app-based tools for remote hearing assessment: a scoping review. Int J Audiol. (2022):1–14. 10.1080/14992027.2022.2075798 35678292

[39] Habib AR Kajbafzadeh M Hasan Z Wong E Gunasekera H Perry C Artificial intelligence to classify ear disease from otoscopy: a systematic review and meta-analysis. Clin Otolaryngol. (2022) 47(3):401–13. 10.1111/coa.13925 35253378 PMC9310803

[40] Forde CT Dimitrov L Doal S Patel J Clare D Burslem M Delivery of remote otology care: a UK pilot feasibility study. BMJ Open Qual. (2022) 11(1):e001444. 10.1136/bmjoq-2021-001444 35135752 PMC8830265

[41] Paglialonga A Cleveland Nielsen A Ingo E Barr C Laplante-Lévesque A. Ehealth and the hearing aid adult patient journey: a state-of-the-art review. Biomed Eng Online. (2018) 17(1):101. 10.1186/s12938-018-0531-3 30064497 PMC6069792

