# Supplementary material for: Digital vs. physical ear-nose-and-throat specialist assessment screening for complicated hearing loss and serious ear disorders in hearing-impaired adults prior to hearing aid treatment: a randomized controlled trial
Source: Front Digit Health. 2023 Jun 8;5:1182421. doi: 10.3389/fdgth.2023.1182421 (PMC10285396; doi:10.3389/fdgth.2023.1182421)
Supplement: Supplementary file 1 [file Table1.docx]

**SUPPLEMENTAL MATERIAL**

APPENDIX OF COLLABORATORS AND CONTRIBUTORS

**The InHEAR trial steering committee:**

Peter Munch Jensen (PMJ) and Søren Jakobsen (SJ), Danish Health Data Authority

Lone Kaalund Thiel (LKT), Jørgen Hougaard (JH), and Helene Højberg Thomsen (HHT), IT and Digitalisation, the North Denmark Region

Henrik Jacobsen (HJ) and Lene Dahl Siggaard (LS), the Department of Otorhinolaryngology and Audiology, Aalborg University Hospital, Aalborg

Sofie Vennike, the Regions of Denmark

Nina Bergstedt, the Danish Ministry of Health

**The InHEAR trial management group:**

HJ, LDS, Dan Dupont Hougaard, and Morten Høgsbro, the Department of Otorhinolaryngology and Audiology, Aalborg University Hospital, Aalborg

**The InHEAR trial supporting committee:**

Majbritt Garbul Tobberub, the Danish Association of the Hard of Hearing

Michael Bille, the Danish Society of Otolaryngology (DSOHH)

Thomas Qvist Barrett, the Danish ENT Specialists’ Organization (DOENHO)

Jesper Yde, the Danish Association of Medical Audiology (DMAS)

Peter Andreasen, the Danish Association of Private Audiology Clinics (PAKS)

Karen Wibling Solgård (KWS), Oticon Denmark

Camilla Vallentin Kristensen, Zealand Hearing Rehabiliation Center, Region Zealand

Marie L Kamp Gonzales, Center for Special Education for Adults (DSV), Copenhagen Municipality

Gert Ravn, FORCE Technology Lab

Martin Bredgaard Sørensen, the Regions of Denmark

PMJ and SF, the Danish Health Data Authority

HJ and LDS, the Department of Otorhinolaryngology and Audiology, Aalborg University Hospital, Aalborg

LKT, JH and HHT, IT and Digitalization, the North Denmark Region

Bent Bilstrup and Christian Gasser, Lakeside A/S

**The Danish Supplier Association for hearing aids:**

KWS, Oticon Denmark

Nicolai Bisgaard and Ivar Thomsen, GN Hearing Denmark

Martin Jensen and Peter Rekling, WS Audiology

Thomas Agerskov, SONOVA

**Participant and trial management centers and collaborators:**

Staff and personnel at the Department of Otorhinolaryngology and Audiology, Aalborg University Hospital, Aalborg, Denmark, and at the four affiliated public hearing rehabilitation clinics in Frederikshavn, Hjørring, Thisted and Hobro in the North Denmark Region.

The administration and personnel at the participating private hearing rehabilitation clinics in the North Denmark Region: AudioNova in Aalborg and Hobro, Dansk HøreCenter in Aalborg and Hjørring, Din Hørespecialist in Aalborg and Frederikshavn, Audika in Nykøbing-Mors and Thisted, Høreapparatet in Aalborg and Aars, Budolfi Høreklinik in Aalborg and Thisted, and VelLyd in Aalborg.

The doctors and personnel at the participating private ENT specialist clinics: The Budolfi ENT specialist clinic in Aalborg, Thisted, and Nykøbing-Mors.

**The four digital assessors:**

Casper Søndergaard, Department of Otorhinolaryngology and Audiology, Aalborg University Hospital, Aalborg, Denmark.

Jesper Hvass Schmidt, Department of Otorhinolaryngology and Audiology, Odense University Hospital, Odense, Denmark

Rikke Haahr, private ENT specialist clinic, Horsens, Denmark

Malene Sine Rokkjær, private ENT specialist clinic, Aarhus, Denmark
